# *Srd5a1* is Differentially Regulated and Methylated During Prepubertal Development in the Ovary and Hypothalamus

**DOI:** 10.1210/jendso/bvad108

**Published:** 2023-08-16

**Authors:** Ben Bar-Sadeh, Lilach Pnueli, Sarai Keestra, Gillian R Bentley, Philippa Melamed

**Affiliations:** Faculty of Biology, Technion-Israel Institute of Technology, Haifa 32000, Israel; Faculty of Biology, Technion-Israel Institute of Technology, Haifa 32000, Israel; Faculty of Biology, Technion-Israel Institute of Technology, Haifa 32000, Israel; Department of Anthropology, Durham University, Durham, DH1 3LE, UK; Department of Anthropology, Durham University, Durham, DH1 3LE, UK; Faculty of Biology, Technion-Israel Institute of Technology, Haifa 32000, Israel

**Keywords:** 5α reductase-1, Srd5a1, hypothalamus, ovary, methylation, epigenetic

## Abstract

5α-reductase-1 catalyzes production of various steroids, including neurosteroids. We reported previously that expression of its encoding gene, *Srd5a1,* drops in murine ovaries and hypothalamic preoptic area (POA) after early-life immune stress, seemingly contributing to delayed puberty and ovarian follicle depletion, and in the ovaries the first intron was more methylated at two CpGs. Here, we hypothesized that this CpG-containing locus comprises a methylation-sensitive transcriptional enhancer for *Srd5a1*. We found that ovarian *Srd5a1* mRNA increased 8-fold and methylation of the same two CpGs decreased up to 75% between postnatal days 10 and 30. Estradiol (E_2_) levels rise during this prepubertal stage, and exposure of ovarian cells to E_2_ increased *Srd5a1* expression. Chromatin immunoprecipitation in an ovarian cell line confirmed ESR1 binding to this differentially methylated genomic region and enrichment of the enhancer modification, H3K4me1. Targeting dCas9-DNMT3 to this locus increased CpG2 methylation 2.5-fold and abolished the *Srd5a1* response to E_2_. In the POA, *Srd5a1* mRNA levels decreased 70% between postnatal days 7 and 10 and then remained constant without correlation to CpG methylation levels. *Srd5a1* mRNA levels did not respond to E_2_ in hypothalamic GT1-7 cells, even after dCas9-TET1 reduced CpG1 methylation by 50%. The neonatal drop in POA *Srd5a1* expression occurs at a time of increasing glucocorticoids, and treatment of GT1-7 cells with dexamethasone reduced *Srd5a1* mRNA levels; chromatin immunoprecipitation confirmed glucocorticoid receptor binding at the enhancer. Our findings on the tissue-specific regulation of *Srd5a1* and its methylation-sensitive control by E_2_ in the ovaries illuminate epigenetic mechanisms underlying reproductive phenotypic variation that impact life-long health.

Early-life stress has numerous well-recognized adverse effects on life-long health. Reproductive function is particularly sensitive, and changes in pubertal timing, hormone levels, menstrual/estrous cycles, and even the size of the ovarian reserve are often evident following stressful events experienced in childhood [1-6]. Such outcomes impact not only fertility, but also reproductive longevity, with consequences for postmenopausal health and susceptibility to noncommunicable disease [7, 8]. We have previously reported that prepubertal immunological stress in a mouse model delayed puberty and led to faster depletion of the ovarian follicle pool, seemingly because of reduced expression of the *Srd5a1* gene in the preoptic area (POA) of the hypothalamus and in the ovaries [9]. We also demonstrated causality for the role of reduced *Srd5a1* expression in the altered reproductive phenotype through inhibition of 5α-reductase-1, which not only reduced GnRH release and mRNA levels in cultured cells, but also delayed pubertal onset in mice [9].

The *Srd5a1* gene encodes the steroidogenic enzyme, 5α-reductase-1, which is responsible for conversion of testosterone to DHT required for healthy ovarian follicle growth [10]. In the brain, this enzyme catalyzes the production of neurosteroids, converting deoxycorticosterone and progesterone to their 5α-reduced forms, which are subsequently converted to tetrahydrodeoxycorticosterone (3α,21-dihydroxy-5α-pregnan-20-one) and allopregnanolone (5α-pregnane-3α-ol-20-one). These neurosteroids act on multiple cell types to regulate the hypothalamic-pituitary-adrenal and gonadal axes, playing important roles in the stress response and likely also in early life programming of these two endocrine axes [1, 9, 11-14].

Our previous study reported that the promoter of *Srd5a1*, which is encompassed by a CpG island, is completely unmethylated in mouse ovaries, but its “shore” in the 5′ end of the *Srd5a1* first intron was more methylated in the ovaries of mice after early-life immune stress than in controls, correlating with its reduced expression. Such shores, at the margins of CpG islands, are regions of lower CpG density, the methylation of which is usually conserved across species, tissue, or cell type, and is closely associated with transcriptional repression [15, 16]. Moreover, most changes in methylation during development and reprogramming occur specifically in these regions of lower CpG density, strongly pointing to a functional modification. Strikingly, in our previous work, we also saw increased methylation at the orthologous genomic region in buccal DNA of Bangladeshi women who had grown up in Bangladesh, where immunological challenges in early-life are relatively high, compared with Bangladeshi migrant women who had grown up in the UK, with better health care and fewer disease exposures in childhood [9]. Notably, this orthologous region harbors several single-nucleotide polymorphisms associated with altered reproductive function in women [17]. Moreover, the women who had spent the first years of their lives in Bangladesh had a shorter reproductive lifespan and lower age-matched ovarian reserve, which were also associated with the higher childhood disease loads in Bangladesh [18-21].

An additional study on the same populations of Bangladeshi women indicated faster rates of epigenetic aging among those who had grown up in Bangladesh compared to migrant Bangladeshi women who grew up in the United Kingdom. Furthermore, based on concordant DNA methylation at the *LHCGR/LHR* locus, the methylation appeared to be more stable [22]. DNA methylation is responsive to multiple external and internal signals and stressors, and altered methylation signatures at key regulatory regions of the genome can mediate long-term effects on gene expression, profoundly affecting physiological function [23-28]. We thus hypothesized that the region of the *Srd5a1* first intron where we found differential methylation acts as a transcriptional enhancer that is regulated by DNA methylation to control *Srd5a1* expression levels. We observed dynamic but distinct changes in *Srd5a1* expression levels and methylation at this intronic enhancer in the ovaries and hypothalamic POA across the lifespan and describe cell-specific regulatory mechanisms of glucocorticoid-activated repression of *Srd5a1* in the POA, and its methylation-sensitive stimulation by estradiol (E_2_) in the ovary.

## Materials and Methods

### Mice

All mice (inbred C57BL/6) were held and handled humanely after protocol approval by the Technion Committee for the Supervision of Animal Experimentation and in accordance with their guidelines. Following euthanasia, brains of these mice were removed and, as previously described [9], placed ventral side up into a matrix (RWD-800-00149-00, model 68713: RWD Life Sciences) for coronal sectioning of the tissue (ie, parallel to the central line of the brain) using a razor blade. The sections located between 5 and 6 mm (or 4.5-5.5 mm for the 7- to 10-day-old mice) contain the POA (Allen Brain Atlas: https://mouse.brain-map.org/) and these were collected for further processing. Two 1-mm holes were punched in these sections, centering on the coordinates −0.465 or 0.465; 1.536; −2.37 mm from the bregma; this sectioning should include harvest of the vascular organ of the lamina terminus, but not the paraventricular hypothalamic nucleus. These two punched tissue specimens from the same mouse were then combined into a single tube to which 1-mL TRIzol was added for DNA and/or RNA extractions. Brain and ovarian tissues from sexually mature females (ages noted in figure legends) were collected in estrus, verified by vaginal smears.

### Quantitative Polymerase Chain Reaction

RNA was isolated with TRIzol, treated with DNase I, and digested and cleaned using R1014 RNA Clean & Concentrator-5 kit (Zymo Research). The cDNA was synthesized using qScript Flex cDNA kit (95049 Quanta) with oligo dT, and real-time quantitative polymerase chain reaction (qPCR) carried out using PerfeCTa SYBR Green FastMix (Quanta). Amplicon levels were quantified using standard curves and normalized to levels of *Rplp0*, all as previously reported [29]. Primers are listed in Table 1.

**Table 1.** Primers

Primer number	Gene	position relative to transcriptional start site	Sequence
**qPCR**			
#184	mRPLP0	140 F	GCGACCTGGAAGTCCAACTA
#185	hRPLP0	240 R	ATCTGCTTGGAGCCCACAT
#1493	Srd5a1	755 F	GAATATGTATCTTCAGCCAAC
#1494	Srd5a1	925 R	GGTAATCTTCAAACTTCTCG
#1891	Gnrh	67F	GATCCTCAAACTGATGGCCG
#1892	Gnrh	271R	CTCCTCGCAGATCCCTGAG
#1931	Fkbp5	1479F	GAGTCCAAAGCCTCAGAGTC
#1932	Fkbp5	1696R	GCCAACACCTTCTCGAAGTC
#1672	Greb1	591F	GCCGAGCAGACAATGAGGAA
#1673	Greb1	806R	CAGGCTGGGAGACTTAGCAC
**qPCR following ChIP**			
#2656	Srd5a1	−200F	GTGCTCCGCTGTGGCGCTGA
#2657	Srd5a1	−65R	AGGGCGCCTTAGTCTCGAGC
#2560	Srd5a1	−384F	TGGACGACCTGATCGTAGC
#2561	Srd5a1	−228R	GCCTACACAGCAAAGACCC
#2297	Srd5a1	12F	GTATCTTCTGGTGGTGCTAG
#2367	Srd5a1	150R	GCCACATATAAGCTCAGGAG
#2673	Srd5a1	223F	GATGCGCTAGTCTACCTGG
#2674	Srd5a1	371R	GAAGGCAGCTCCTGTAGGA
#2272	Srd5a1	483F	GTCTTCCCTCCTGCGCTTG
#2273	Srd5a1	636R	GAAATCCGGACCACTGTGC
#2664	Srd5a1	676F	GCGATGCCATCCAAGCTGC
#2665	Srd5a1	828R	CTCTGAAATTGCTCCAGTCC
#2287	Srd5a1	862F	CTTTCCCAGGAGGTGTTATG
#2652	Srd5a1	970R	GGGTCAGTTAAAGATAAGACC
#2288	Srd5a1	1095R	GACTTTCCCATGTCCCAAATG
#2233	Srd5a1	1152F	GTTGTGTTAATAGCCTCTGC
#2234	Srd5a1	1322R	GCTGTTACACAGAGAAACTCG
#2438	Srd5a1	3413F	GTTAAACCCTCCGAGATAGAC
#2439	Srd5a1	3565R	CCCACTCTGTGTCACTAAGTG
#2554	Srd5a1	8465F	CTCAAAGTCCCCACTCTAG
#2555	Srd5a1	8679R	CTTTCTCATGGATGGATCAC
#2496	Srd5a1	12621F	GGCAAGTAACAGAGGAAGAG
#2497	Srd5a1	12773R	CCCTTCACTCTGCTCTTACA
#2440	Srd5a1	18043F	GCGTGGTAGGGGACAAGAG
#2441	Srd5a1	18197R	CCACATCTGGAATCAGGTAC
#2498	Srd5a1	21898F	GAGGTTTCCATAAGGGAGCA
#2499	Srd5a1	22054R	ATGAAGTGGCAACGCCTTTC
#2233	Fkbp5	1479F	GAGTCCAAAGCCTCAGAGTC
#2234	Fkbp5	1696R	TGGACGACCTGATCGTAGC
#1404	Pgr	459F	AGGACAGGAGCTGACCAAGA
#1405	Pgr	640R	AGTCATGACGACCCAAGCTC
**PCR on bisulfite converted DNA**			
#1628	Srd5a1 BS	−123F	AAGGAGTTTTTAGTTAATGTGTGTAG
#1629	Srd5a1 BS	62R	AAACACAAACTAACACCACCAAAA
#1822	Srd5a1 BS	−301F	GGGTTAGATTGTGGAGGGG
#1823	Srd5a1 BS	123R	CAAAACAACCCACAAAAACCAAC
#1824	Srd5a1 BS	845F	GTGTGAGATGGTATGAATTTTTTTT
#1825	Srd5a1 BS	871F	GGAGGTGTTATGTGAAAAATGTTT
#1826	Srd5a1 BS	1081R	CCAAATATCACAAAACTCAACTTC
#1827	Srd5a1 BS	1148R	CATTCTCCCAACCTCTCTAAAAA
#2238	Srd5a1 BS	597F	GTGTTTGGTTAGGGATAGTGGT
#2217	Srd5a1 BS	875R	CCTCCTAAAAAAAATTCATACCAT
#2215	Srd5a1 BS	605F	TTAGGGATAGTGGTATAGTGGTT
#2216	Srd5a1 BS	847R	CACAAAAAAAACAAAACATCTCTAAA
#1881	Srd5a1 BS + adapter—MiSeq	871F	TCGTCGGCAGCGTCAGATGTGTATAAGAGACAGGGAGGTGTTATGTGAAAAATGTTT
#1882	Srd5a1 BS + adapter—MiSeq	1081R	GTCTCGTGGGCTCGGAGATGTGTATAAGAGACAGCCAAATATCACAAAACTCAACTTC
#2258	Srd5a1 BS + adapter—MiSeq	605F	TCGTCGGCAGCGTCAGATGTGTATAAGAGACAGTTAGGGATAGTGGTATAGTGGTT
#2259	Srd5a1 BS + adapter—MiSeq	847R	GTCTCGTGGGCTCGGAGATGTGTATAAGAGACAGCACAAAAAAAACAAAACATCTCTAAA
**sgRNAs**			
#2460	Srd5a1 sgRNA5	849F	CACCGAGATGGTATGAATCTTTCCC
#2461	Srd5a1 sgRNA5	869R	AAACGGGAAAGATTCATACCATCTC
#2462	Srd5a1 sgRNA6	1105F	CACCGCTCTGATCCTAAAGTATTCA
#2463	Srd5a1 sgRNA6	1125R	AAACTGAATACTTTAGGATCAGAGC

Abbreviations: PCR, polymerase chain reaction; qPCR, quantitative polymerase chain reaction.

### Methylation Analysis

DNA was extracted from tissues using TRIzol, after taking the upper phase for RNA extractions. The DNA was then cleaned using the Quick-DNA Miniprep Plus Kit (D4068; Zymo) before bisulfite conversion using the EZ-DNA Methylation-Gold Kit (D5005 Zymo) and two rounds of PCR amplification (nested, with outer and inner primers: Table 1) using Red Load Taq Master (Larova). For analysis of untreated cell lines, where there is less variation in methylation levels between samples, the amplicons were purified (DNA Clean and Concentrator kit D4004; Zymo) and cloned into pGEM-T-easy, before inserts from 7 to 20 randomly selected clones were sequenced and analyzed as previously described [30]. For tissue samples from the mice, deep sequencing was performed: after bisulfite conversion, the region of interest was cleaned and amplified with the listed primers (Table 1). An additional 8 to 12 cycles of PCR (30 seconds each, at 65 °C) were then performed using KAPA HiFi HotStart Ready mix X2 (Roche), with a different combination of Illumina Nextera XT indexes (10 µM) for each sample. Samples were cleaned with PCR purification kit (Zymo) between each PCR round. After addition of 50% Phi-X, these libraries were deep-sequenced by 150-bp paired-end sequencing on Mi-seq (Illumina), at the Technion Genome Center. The % methylation levels represent the relative number of cytosines found methylated out of the total number sequenced at the same site.

### Cell Culture

The GT1-7 mouse hypothalamic GnRH neuronal cell line was cultured with high glucose DMEM containing 10% fetal bovine serum (FBS), 1% penicillin-streptomycin, sodium pyruvate, and sodium bicarbonate (all from Biological Industries, Beit Haemek), maintained at 37 °C with 5% CO_2_ at 50% to 90% confluency, passaging 1 to 2 times a week. The media was replaced with the same media but containing charcoal-stripped FBS, 24 hours before and during treatments with E_2_ or dexamethasone (Dex; Sigma), as described. The murine KK-1 granulosa cell line (a gift from Ilpo Huhtaniemi, Imperial College, UK) was cultured as reported [31], maintained at 37 °C with 5% CO_2_ at 30% to 80% confluency, passaging 2 to 3 times a week. Steroid treatments were performed in each cell line across several doses of the steroid (in this or our previous study [9]), which were chosen initially based on those commonly used in similar cell culture gene expression analysis and specifically in these cell types (eg [32-34],). Subsequently, after the dose-response analysis indicated which doses elicited effects on gene expression, the optimal dose (lowest dose showing most significant effect) was chosen for future experiments, and control genes or loci were included to confirm responses to these treatments. Cells are tested regularly for mycoplasma and identity authenticated through hormone responsiveness.

### Primary Culture

On harvest, ovaries were transferred immediately into Hanks’ Balanced Salt Solution that lacked Mg and Ca (Biological Industries). The Hanks’ Balanced Salt Solution was then replaced with papain solution [35] for 20 minutes at 37 °C to loosen cell-cell interactions. Subsequently, the papain solution was replaced with growth media containing charcoal-stripped FBS, as for KK-1 cells, and the tissue was pipetted several times to separate the cells (mostly granulosa), which were then seeded into a 96-well plate. Approximately 24 hours later, fresh media with E_2_, or ethanol as a vehicle control, was added for 24 hours before RNA extraction.

### Site-directed Manipulation of the DNA Methylation

KK-1 cells were transfected with pCMV-dCas9-D3A plasmid (Addgene #78256 [36]) to stably express catalytically dead Cas9 (dCas9) fused with the catalytic domain of DNMT3A and FLAG tag peptide. The plasmid was first linearized (*NotI HF* [R3189S BioLabs] for 1 hour at 37 °C), and 1 µg/mL plasmid transfected using Lipofectamine 3000 (L3000015, Invitrogen) to cells at 70% to 80% confluency in a 35-mm plate. The transfected cells were selected with 600 µg/mL Bleomycin (Zeocin ant-zn-1, InvivoGen) for 2 to 3 weeks, and clones tested for DNMT3A mRNA by qPCR and protein by western blot with FLAG peptide monoclonal antibody (Sigma F3165, RRID:AB_259529).

These cells were transiently transfected with gRNA (planned with *benchling.com*) for recruitment of the dCas9-DNMT3A to the 5′ and 3′ ends of *Srd5a1* enhancer. Each annealed gRNA was ligated into linearized (*Esp3I* [R0734S, BioLabs] for 2 hours at 37 °C) pSB700 plasmid (#64046 Addgene) modified to express mCherry fluorescent protein, with T4 DNA ligase (M180B, Promega) in T4 Rapid Ligation Buffer X2 (C6711, Promega), for 60 minutes at room temperature. Cells were transfected with this plasmid as described previously and after ∼48 hours, FACSAria-IIIu cell sorter separated the mCherry-positive and negative cells. These cells were seeded on a 24-well plate with phenol red-free DMEM/F12 medium containing charcoal-stripped FBS. After 24 hours, the cells were exposed to 10 nM E_2_ for 24 hours, followed by RNA and DNA extractions.

For the site-directed demethylation, GT1-7 cells were cotransfected with TETv4 plasmid (Addgene #167983) and pSB700 containing the same gRNAs as previously, or empty pSB700 as control. After ∼48 hours, the cells that were successfully transfected for both plasmids (expressing mCherry and BFP) were collected using the FACSAria-IIIu cell sorter. These cells were seeded on a 96-well plate with charcoal stripped serum-containing medium and harvested 24 hours later.

### Chromatin Immunoprecipitation

Chromatin immunosuppression (ChIP) was carried out as described [37, 38] after formaldehyde cross-linking, sonicated to an average of 200-bp fragments and with the following antibodies: ESR1 (Abcam 32063, RRID:AB_732249), GR (Abcam 3671, RRID:AB_2236351), H3K4me1 (Abcam 8895, RRID:AB_306847), and FLAG peptide (Sigma F3165, RRID:AB_259529). The DNA was purified and regions amplified by qPCR (as detailed earlier; primers in Table 1) from immunoprecipitation samples and from the input to which the immunoprecipitation amplicon levels were normalized.

### Statistical Analysis

All data are from multiple biological repeats (n-value), which were assayed individually. Results are shown as mean ± standard error of the mean. Parametric data were analyzed by 2-tailed Student *t*-test or 1-way ANOVA followed by Tukey-Kramer or Bonferroni *t*-test for multiple comparisons. Methylation analysis (% methylation) used Mann-Whitney or Kruskal Wallis, Dunn nonparametric *t*-test. Significance was defined as *P* < .05.

## Results

### *Srd5a1* is Differentially Regulated in the Ovaries and Hypothalamus Across the Lifespan

**Figure 1.**
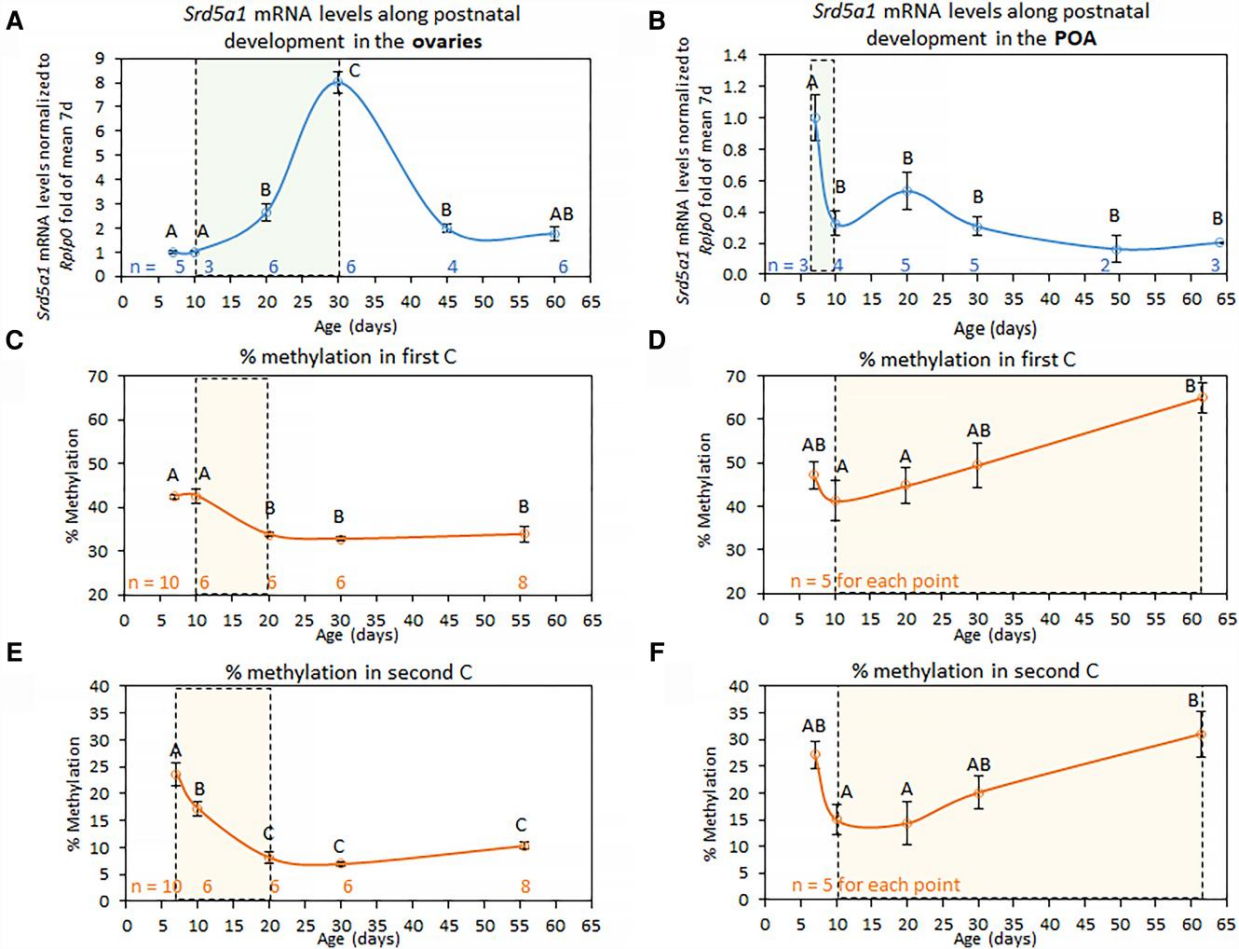
*Srd5a1* is differentially regulated in ovaries and hypothalamus across the lifespan. (A, B) *Srd5a1* mRNA levels in (A) ovaries and (B) the hypothalamic preoptic area (POA) of mice at various ages (from 1 to 4 litters at each time point; for >50 days old, mice in each group were not identical ages and the average age is shown). The mRNA levels were normalized to those of *Rplp0* and are shown relative to levels at the first time point; mean ± standard error of the mean (SEM; some of the ovarian data are from [9]). (C-F) The % DNA methylation (% of cytosines methylated out of the total number sequenced at the same site) measured by bisulfite conversion followed by MiSeq deep sequencing at the (C, D) first and (E, F) second CpG in (C, E) ovaries and (D, F) POA. In all graphs, n-values at each point are shown; *P* > .05 (ANOVA, Tukey-Kramer *t*-test) for groups sharing the same letter. Shaded boxes mark periods of significant change.

Having found previously that *Srd5a1* expression is reduced in the POA and ovaries following early-life immunological challenge and that this was associated in the ovaries with increased methylation at two CpGs in the first intron [9], we looked first at how *Srd5a1* mRNA and methylation levels at these loci vary across early development under normal conditions. We found that *Srd5a1* expression levels changed across the prepubertal period, in a manner that differed markedly in the two tissues. Ovarian *Srd5a1* mRNA levels increased dramatically between mice at postnatal day (PND) 10 and 30 but were much lower in postpubertal mice examined at PND 45 (Fig. 1A). However, in the POA, they decreased sharply in mice aged between PND 7 and 10 and appeared relatively consistent in the mice older than this (Fig. 1B).

Levels of methylation at these two intronic CpGs (CpG1 and CpG2) were measured by bisulfite conversion and high-throughput sequencing (MiSeq). In the ovaries, a significant drop in DNA methylation (between PND 7 and 20) was seen before the increase in expression of *Srd5a1*: at the second CpG (CpG2) the levels dropped more dramatically from 24% to 7%, and at both CpGs they appeared to remain consistent thereafter (Fig. 1C and 1E). In the POA, there appeared to be some drop in methylation between PND 7 and 10, though this was not statistically significant, perhaps because of the small sample size. It is clear, however, that the drop in *Srd5a1* expression in the POA of the mice at this early neonatal stage was not negatively correlated with change in the methylation levels. The methylation was generally higher in the POA than in the ovaries, and from PND 10, levels at both CpGs appeared to increase with aging (Fig. 1D and 1F). *Srd5a1* is thus clearly regulated differently in these two tissues.

### E_2_ Increases *Srd5a1* Expression in Ovarian Granulosa Cells, and Estrogen Receptor-1 (ESR1) Binds the Locus of the Differentially Methylated CpGs at a Transcriptional Enhancer

**Figure 2.**
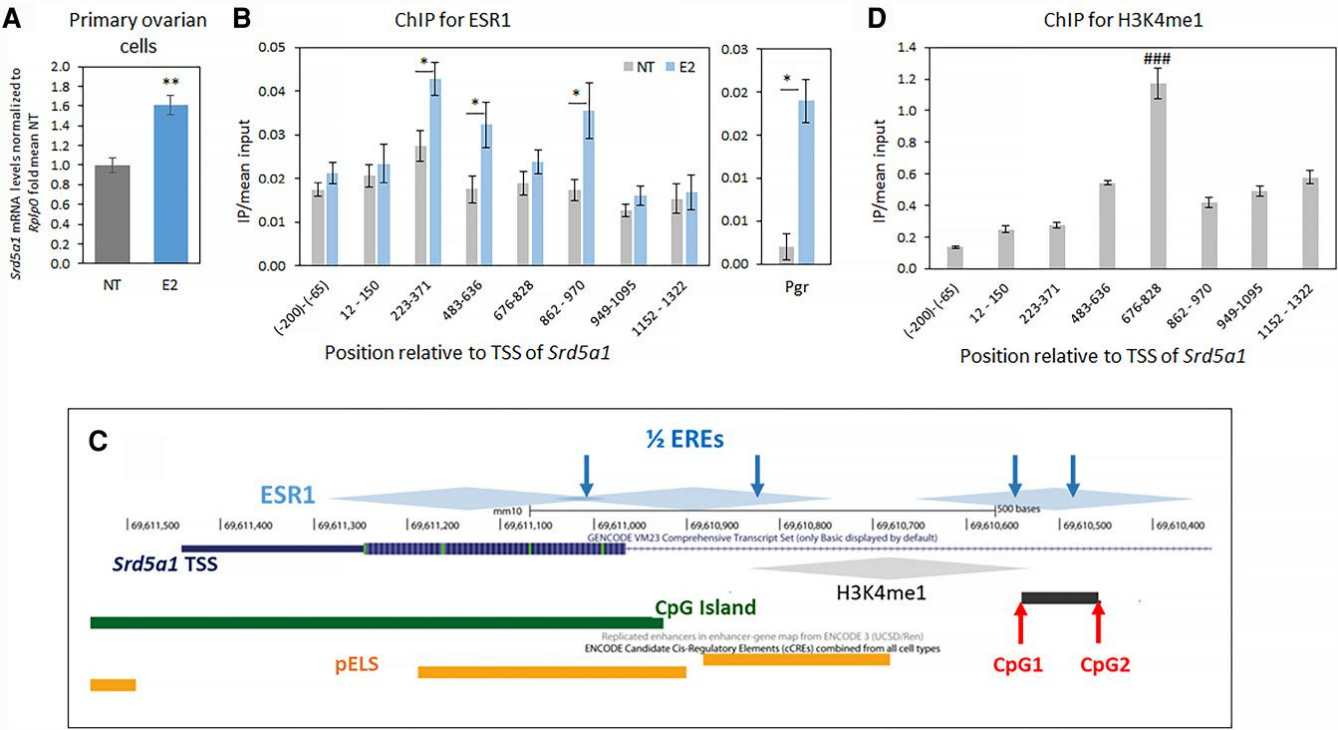
E_2_ increases *Srd5a1* expression in ovarian granulosa cells, and estrogen receptor (ESR1) binds the locus of the differentially methylated CpGs at a transcriptional enhancer. (A) *Srd5a1* mRNA levels in mouse ovarian primary cell culture (mice were 30 days old) after exposure to E_2_ (100 nM) for 24 hours; mean ± SEM, n = 3, 4; **P < .01. (B) Chromatin immunoprecipitation (ChIP) for ESR1 in murine ovarian granulosa KK-1 cells with or without 2 hours exposure to E_2_ (10 nM) followed by qPCR for loci across the 5′ end of the gene and first intron, with *Pgr* as positive control. IP/input levels are mean ± SEM (n = 7, except at 223-371, 676-828, and *Pgr* where n = 4). Student *t*-test compared treated and nontreated groups, *P < .05. (C) Schematic (adapted from UCSC genome browser; http://genome.ucsc.edu) showing the locus of the 5′ end of the *Srd5a1* gene (in continuous dark bar: UTR [thin bar], first exon [thick bar], and part of first intron [thin line]) in the mouse genome, with the location of CpG1 and CpG2, and ½ ERE motif sites marked (arrows). The CpG island and several regions identified by ENCODE as proximal enhancer-like sequences (pELS) are shown, as well as the regions we found enriched for ESR1 or H3K4me1 in KK-1 cells (diamonds, centered on the amplicon center and in accordance with the resolution determined by sonication). (D) ChIP assay for H3K4me1, performed and presented as in Fig. 2B (n = 4); ^###^*P* < .001 (ANOVA, Tukey-Kramer *t*-test) compared with all other means.

The dramatic increase in ovarian *Srd5a1* expression between PND 10 to 30 suggested that the gene might be regulated by gonadal steroids, supported by the fact that E_2_ activity has been shown already by PND 15 [39, 40], and our previous observations that E_2_ induced an increase in *Srd5a1* mRNA levels in the KK-1 ovarian granulosa cell line [9]. Granulosa cells are the most abundant cell type in the ovary and the main cell type that expresses *Srd5a1* (www.proteinatlas.org/ENSG00000145545-SRD5A1/single+cell+type/ovary [41, 42]), and we confirmed the *Srd5a1* response to 24 hours E_2_ in primary ovarian cells from 30 day-old mice (Fig. 2A). To examine further the mechanisms through which E_2_ regulates *Srd5a1*, we performed ChIP for ESR1 in the same KK-1 ovarian granulosa cells. In E_2_-treated cells, ESR1 was enriched in the region of the first exon-intron boundary in accordance with the presence of two half estrogen response elements (EREs: consensus TGACC and nonconsensus GGGCA [43]; Fig. 2B and 2C). ESR1 was also enriched further downstream in the intron, where there are two more consensus half-sites, one of which encompasses CpG1, whereas the other is located closer to CpG2 (Fig. 2B and 2C). The orthologous region in the human genome, which includes the differentially methylated CpGs and early menopause-associated single-nucleotide polymorphism identified previously [9], contains two sequences predicted (by JASPAR) to function as EREs.

In the human genome, this orthologous region is indicated (GeneHancer [44], visualized in the UCSC genome browser: http://genome.ucsc.edu) to act as a transcriptional enhancer, and ENCODE data show it is highly enriched with H3K4 monomethylation (H3K4me1) [9], a typical histone modification of transcriptional enhancers [45]. In the mouse genome, this locus is enriched with ENCODE candidate *cis*-Regulatory Elements comprising a proximal enhancer-like signature (Fig. 2C). We thus performed ChIP for this histone modification in the mouse ovarian granulosa cell line, which revealed that H3K4me1 is strongly enriched at this locus (Fig. 2D), indicating that it indeed likely functions as a transcriptional enhancer in these cells.

### In Ovarian KK-1 Granulosa Cells, Methylation of the Intronic Enhancer CpG2 Prevents E_2_ Stimulation of *Srd5a1* Expression

**Figure 3.**
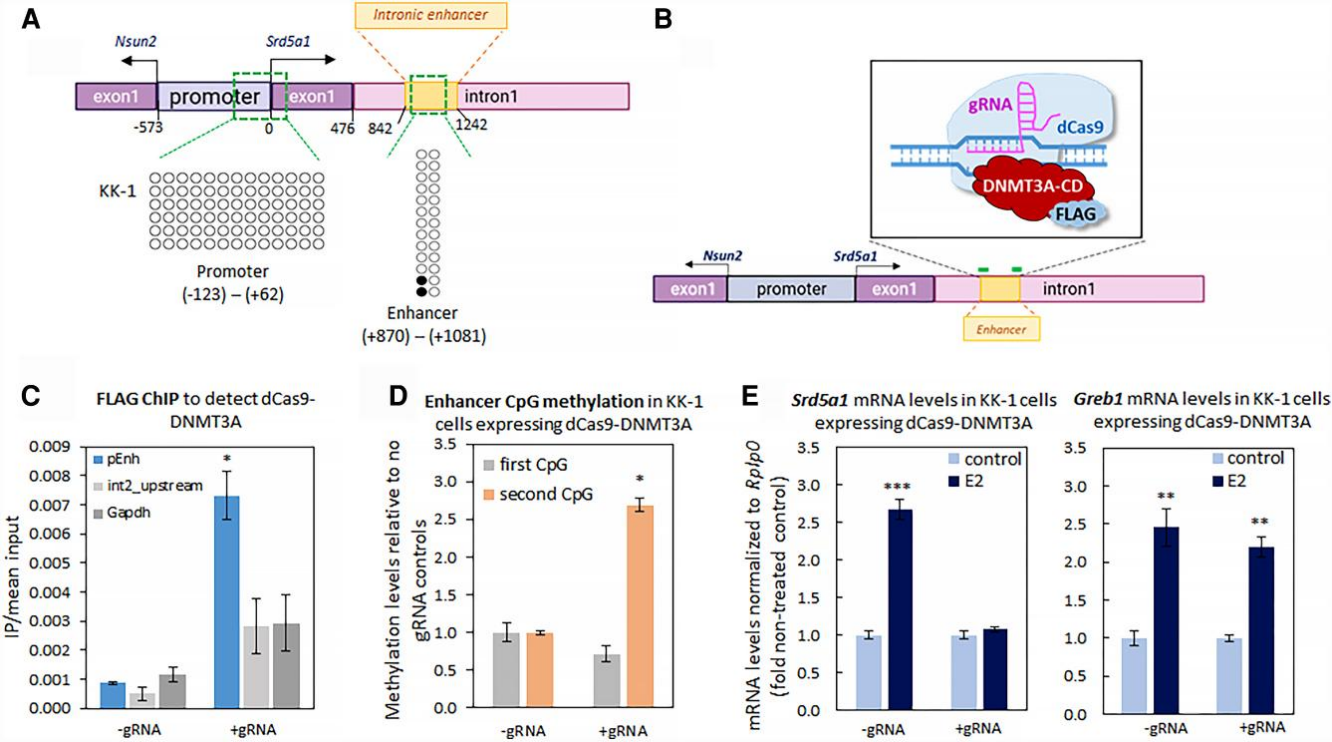
In ovarian KK-1 granulosa cells, methylation of the intronic enhancer CpG2 prevents E_2_ stimulation of *Srd5a1* expression. (A) DNA methylation (bisulfite conversion and sequencing) at the *Srd5a1* promoter (−123 to +62 bp) and intronic enhancer (+870 to +1081, including CpG1 and CpG2) in ovarian KK-1 cells: each column represents a single CpG site, and each row a repeat; black circles represent CpGs that are methylated and white circles those that are not. (B) Targeted DNA methylation was performed by stable expression of a FLAG-tagged dCas9-DNMT3A catalytic domain, recruited to the enhancer of *Srd5a1* by two site-specific gRNAs (thick green lines). (C) ChIP assay for FLAG peptide in KK-1 cells stably expressing dCas9-DNMT3A-FLAG, after transfection with the gRNAs or empty vector, followed by qPCR for the *Srd5a1* intronic enhancer, an upstream region and *Gapdh* as controls; IP/input levels presented as in Fig. 2B (n = 3). Student *t*-test compared levels in cells with and without transfection of the gRNAs; * < 0.05. (D) Levels of DNA methylation (measured by bisulfite conversion and MiSeq) in these cells at CpG1 and CpG2 of the *Srd5a1* intronic enhancer, shown relative to those in control cells (no gRNAs); mean ± SEM (n = 3). (E) *Srd5a1* and *Greb1* mRNA levels in these cells with or without E_2_ (10 nM, 24 hours). The mRNA levels were analyzed and presented as before (n = 3); **P < .01, ***P < .001 compared with untreated controls.

Given the drop in methylation levels at the two intronic enhancer CpGs in the ovaries before the prepubertal increase in *Srd5a1* expression (Fig. 1), we went on to investigate a possible regulatory role for this methylation. To ascertain the utility of the KK-1 ovarian cell line for this purpose, we first examined the *Srd5a1* proximal promoter (−123 to +62 bp from the transcriptional start site) and enhancer (+870 to +1081 bp from the transcriptional start site), both of which we found to be virtually unmethylated in these cells (Fig. 3A). We next investigated whether inducing DNA methylation at this enhancer in the ovarian granulosa cell line would be sufficient to inhibit *Srd5a1* expression. For this, we used KK-1 cells stably expressing a FLAG-tagged dCas9-DNMT3A catalytic domain, targeted to the enhancer by two site-specific gRNAs (Fig. 3B). Binding of the chimeric protein to this locus was confirmed by ChIP and was seen to be enriched at the enhancer only in the presence of the gRNAs (Fig. 3C). Methylation levels were assessed (by bisulfite conversion and MiSeq), and were increased 2.5-fold at CpG2, whereas CpG1 was not affected (Fig. 3D). Strikingly, this treatment abolished the response of *Srd5a1* to E_2_, whereas that of the control E_2_-activated gene, *Greb1*, was unaltered (Fig. 3E). We have thus established a facilitating role for the demethylation at this site in E_2_ up-regulation of *Srd5a1*.

### In Immortalized GnRH Neuronal GT1-7 Cells, *Srd5a1* mRNA Levels are not Affected by E_2_ Even After Reduction in Methylation at the Intronic Enhancer CpG1

**Figure 4.**
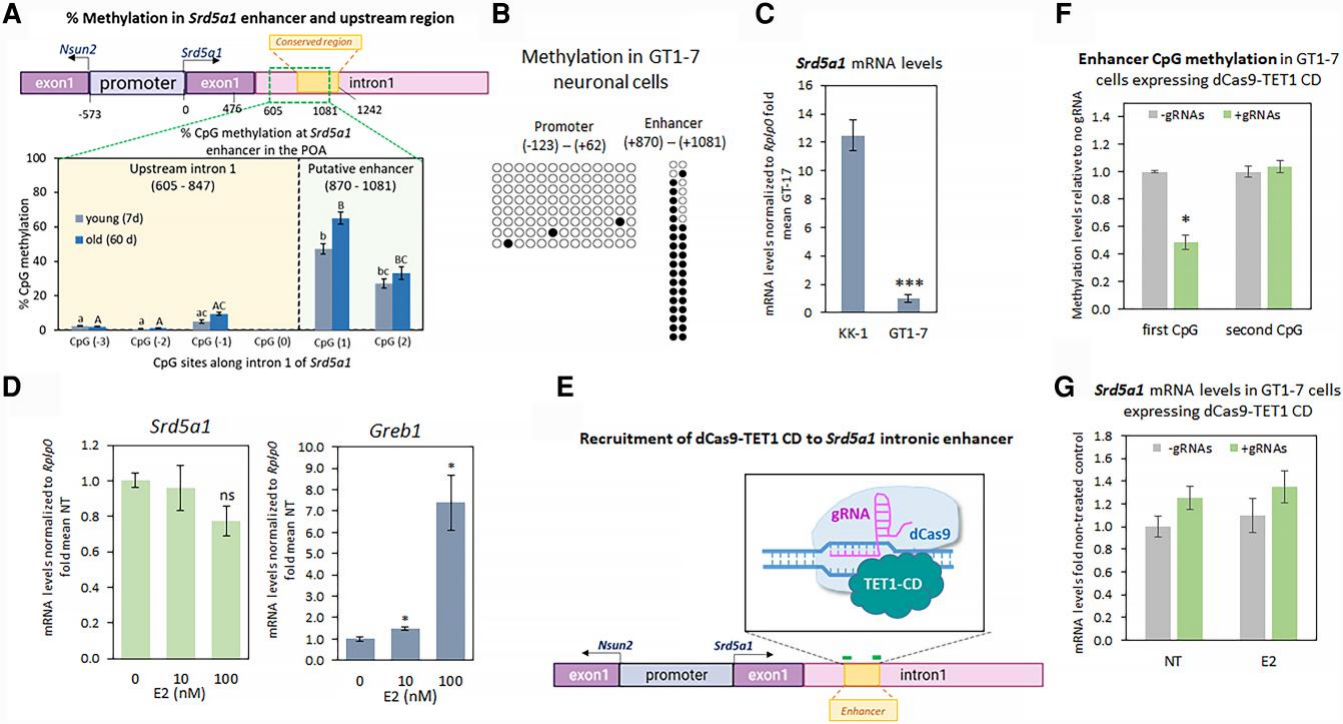
In GnRH neuronal GT1-7 cells, *Srd5a1* mRNA levels are not affected by E_2_ even after reduction in intronic enhancer CpG1 methylation. (A) DNA methylation at the intronic enhancer and ∼300 bp adjacent upstream region in the POA of 7- and 60-day-old female mice was performed and is presented as % methylation, mean ± SEM (n = 4); small or capital letters designate statistical tests for each age group separately (Kruskal Wallis, Dunn test); CpG (0) had no detectable methylation. (B) DNA methylation was assessed at the *Srd5a1* promoter (−124 to +62 bp) and intronic enhancer (CpG1 and CpG2) in GT1-7 cells, as in Fig. 3A and is presented similarly. (C, D) *Srd5a1* mRNA levels in (C) KK-1 and GT1-7 cells, or (D) GT1-7 cells after 10 to 100 nM E_2_ exposure (*Greb1* serves as positive control), measured and presented as before (n = 4); *P < .05, ***P < .001. (E) Targeted demethylation was performed by overexpression of dCas9-TET1 catalytic domain, recruited to the *Srd5a1* enhancer by the same site-specific gRNAs as in Fig. 3B (thick green lines). (F) DNA methylation (bisulfite conversion and MiSeq) at CpG1 and CpG2 of the *Srd5a1* intronic enhancer in the GT1-7 cells expressing the dCas9-TET1. Levels are presented relative to those in control cells (no gRNAs); mean ± SEM (n = 3); *P < .05. (G) *Srd5a1* mRNA levels in similarly transfected cells, with or without exposure to E_2_ (10 nM), presented as before (n = 3); *P* > .05 in *t*-test for all comparisons.

Given the differential expression of *Srd5a1* in the POA and ovaries, we looked for the underlying mechanisms, and first asked whether this enhancer might be methylated at additional sites other than CpG1 and CpG2 in the hypothalamic POA. However, the other four CpGs in the H3K4me1-enriched region (+605 to +847) had very low levels of DNA methylation (<10%) in the POA of both young (7 days) and adult (60 days) mice (Fig. 4A), suggesting an unlikely regulatory role in this context.

Although methylation at the intronic enhancer CpGs in the POA did not appear to be correlated with *Srd5a1* expression across early development (Fig. 1), in light of our findings in the ovaries, we wanted to determine whether methylation of these CpGs had any effect on *Srd5a1* expression in neuronal cells. The GT1-7 cell line is derived from GnRH-producing hypothalamic POA neurons, and *Srd5a1* is expressed in primary GnRH neurons [46]), so we first examined methylation at the *Srd5a1* proximal promoter and enhancer in this cell line. As in the ovarian cell line (Fig. 3A), the *Srd5a1* promoter was practically unmethylated in these neuronal cells, but both enhancer CpGs (CpG1 and CpG2) were highly methylated (Fig. 4B) in line with the findings in the primary cells (Fig. 1). This differential enhancer methylation also correlated with very different *Srd5a1* expression levels that were more than 12-fold higher in the ovarian KK1 cell line than in the neuronal GT1-7 cells (Fig. 4C).

Following our findings in the ovarian cell line, we next examined whether in this estrogen-responsive GT1-7 neuronal cell line, *Srd5a1* mRNA levels might be increased by E_2_. However, no effect on *Srd5a1* was seen, despite a clear increase in expression of the positive control gene, *Greb1* (Fig. 4D). To determine whether a reduction in the enhancer DNA methylation in these cells might be sufficient to allow increased *Srd5a1* expression, we targeted the demethylating dCas9-TET1 catalytic domain [47] to the region with two site-specific gRNAs (Fig. 4E). After 3 days, the methylation levels were reduced by >50% at CpG1 but were unaltered at CpG2 (Fig. 4F). Despite the significant reduction in methylation at CpG1, there was no apparent effect on *Srd5a1* expression, and *Srd5a1* mRNA levels were still not responsive to E_2_ exposure (Fig. 4G).

### The Glucocorticoid, Dexamethasone, Represses *Srd5a1* Expression in GT1-7 Neuronal Cells, and the Glucocorticoid Receptor is Found at the Intronic Enhancer.

**Figure 5.**
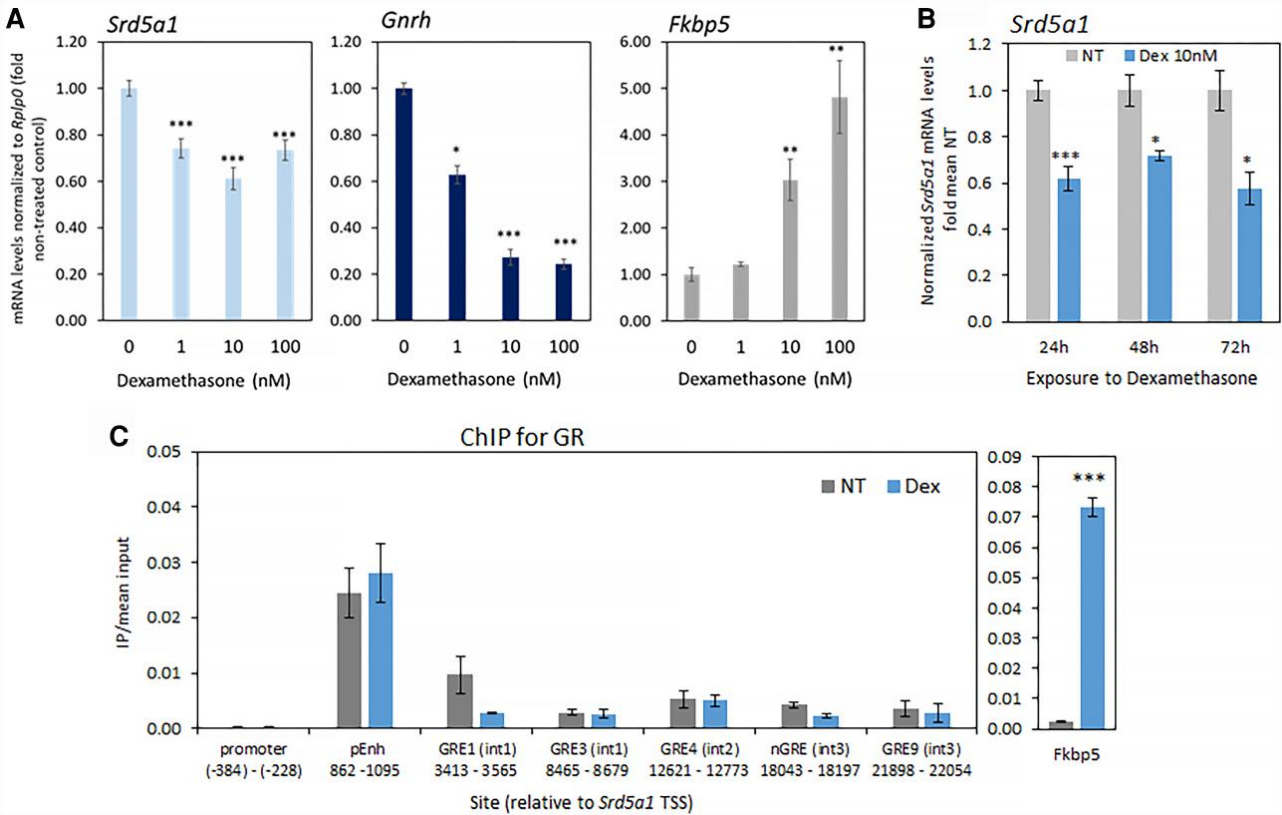
The glucocorticoid, dexamethasone, represses *Srd5a1* expression in GT1-7 neuronal cells, and the glucocorticoid receptor is found at the intronic enhancer. (A, B) *Srd5a1* mRNA levels in GT1-7 cells after (A) 1-100 nM dexamethasone (Dex; n = 6-7) for 24 hours, with *Gnrh* and *Fkbp5* as controls, or (B) 10 nM Dex for 24 to 72 hours (n = 3-4); data analyzed and presented as before. (C) ChIP assay for GR in GT1-7 cells after Dex exposure (10 nM, 24 hours), and qPCR for identification of binding at the *Srd5a1* promoter, enhancer, and additional putative sites (from chip-atlas.org), with *Fkbp5* as positive control, performed and presented as in Fig. 2B (n = 3). Student *t*-test compared treated and nontreated groups; ***P < .001, otherwise *P* > .05.

The levels of both glucocorticoids and the glucocorticoid receptor (GR) are particularly low in the immediate neonatal hyposensitive period, increasing after about PND 8 to 10 [48, 49], with circulating corticosterone levels rising by as much as 300-fold between PND 5 and 15 [50]. This timing corresponds with the dramatic drop in *Srd5a1* levels (Fig. 1B). Moreover, our previous studies found that early-life immune stress (PND 23-30) caused a reduction in POA *Srd5a1* expression [9], indicating a possible role for glucocorticoids in regulating *Srd5a1* in this region of the brain. The GR (or Nr3c1) is expressed in primary GnRH neurons [46], so we treated GT1-7 cells with the synthetic glucocorticoid, Dex, for 24 to 72 hours to assess its effects on *Srd5a1* expression. Dex significantly reduced *Srd5a1* mRNA levels (Fig. 5A and 5B), although the effect was not dose-dependent over 1 to 100 nM (Fig. 5A).

To explore further the effect of glucocorticoids on *Srd5a1*, we also performed ChIP for GR in these neuronal cells to identify the GR-binding sites in this region of the gene. GR was enriched at the *Srd5a1* intronic enhancer, although not detected at various putative GR response elements, including those indicated in other tissues (Chip-atlas.org). Despite Dex treatment strongly increasing GR binding at the control, *Fkbp5* locus, it did not seem to affect GR binding at the *Srd5a1* enhancer (Fig. 5C), suggesting glucocorticoid modification of receptor activity rather than DNA binding at this site. Notably, Dex was seen previously to have no effect on *Srd5a1* expression in KK-1 ovarian cells [9]. Together, these findings support the existence of a cell-specific regulatory mechanism of glucocorticoid-activated repression in the POA, in contrast to the cell-specific stimulatory effects of E_2_ in the ovary.

## Discussion

5α reductase-1 plays a central role in endocrine systems regulating brain function, the stress response and reproduction. This, together with the discovery that the encoding gene, *SRD5A1,* is sensitive to epigenetic modifications, emphasizes the need to understand the molecular mechanisms regulating its expression. Based on our previous findings of altered reproductive function following early-life immune stress [9], we hypothesized that reduced *Srd5a1* expression in the ovaries and hypothalamus is due to increased methylation in the first intron that was evident in the mouse ovarian and women's buccal DNA in the earlier study. We have now identified this locus as a transcriptional enhancer, the activity of which is affected by methylation. Surprisingly, however, we saw very different patterns of *Srd5a1* expression in the ovaries and POA across murine postnatal development.

The finding that *Srd5a1* mRNA levels in the ovaries peaked at PND 30 is similar to reports in male mice in which *Srd5a1* levels in the testes were seen to peak at PND 25 [51]. Although the very low levels of E_2_ in prepubertal mice make accurate measurements difficult, its levels and/or activity are reportedly elevated and play a role in reproductive development already by PND 15 [39, 40]. This, together with the stimulatory effect of E_2_ on *Srd5a1* expression in cultured ovarian cells, suggested that E_2_ might be responsible for the increase in ovarian *Srd5a1* levels at this stage of development. A stimulatory effect of E_2_ on *Srd5a1* expression has been reported in human breast cancer cell lines, mediated though ESR1 binding to an upstream distal enhancer, tethered via additional proteins [52]. We found ESR1 at the novel intronic enhancer, providing an additional locus of activation via a half ERE, suggesting also a tethering mechanism, which has been reported for ESR1 binding in many cellular and genomic contexts [53-55]. The exact mechanisms of ESR1 binding to this *Srd5a1* enhancer, as well as its coactivators and regulatory mechanisms that are often diverse and complex [53, 56-59], have yet to be identified.

DNA methylation has been shown to affect ESR1 binding at numerous genomic loci [60-63] and the fact that the increase in ovarian *Srd5a1* expression in prepubertal mice followed a drop in DNA methylation suggested a possible connection. This connection was confirmed when increased methylation at one of the CpGs abolished the *Srd5a1* response to E_2_. Targeting of DNMT3A to this region increased methylation levels only at the more distal CpG (CpG2), whereas the ERE sites are located a little further upstream and overlap CpG1. However, DNA methylation affects transcription factor (TF) binding through various mechanisms [64]. At the TF binding site, methylation can alter TF binding kinetics, stability and/or its dissociation, which may be position-dependent within the motif [65-68]. Methylated DNA is also recognized by specific methylated DNA-binding proteins which recruit additional chromatin modifying proteins that alter the chromatin landscape. Such changes, including nucleosome positioning and histone variants, would certainly impact binding site accessibility and dynamics [69]. Moreover, methylation alters the mechanical characteristics of DNA, such as its shape, flexibility, and hydration [70-72], any of which would likely affect TF binding in the vicinity.

Epigenetic modifications play various roles in the central control of puberty [73, 74], and methylation levels change across sexual development [75-77], particularly at loci enriched with high-affinity EREs [76]. In patients with breast cancer, the negative correlation between methylation and ESR1 induction of gene expression involved mostly CpGs located at enhancers >1 kbp downstream of the transcription start site [78, 79], corresponding with the location of the intronic enhancer of *Srd5a1*. Our findings suggest that the drop in methylation during early postnatal development is required for E_2_ induction of *Srd5a1* expression in the ovary. This drop in methylation is, however, unlikely sufficient for gene activation, which would depend also on circulating E_2_ levels and activity (eg, as regulated by α-fetoprotein [39]), as well as the expression of ESR1 in granulosa cells of the developing follicles [80].

In contrast with its expression and up-regulation in the ovaries, *Srd5a1* expression was not increased by E_2_ in GnRH neuronal cells, and we did not detect any major changes in its expression in the POA toward puberty when circulating and brain E2 levels are elevated [39, 40]. The only dramatic change evident in POA *Srd5a1* expression levels along the life course was a drop in the neonate, following the stress-hyporesponsive period when circulating glucocorticoid levels start to increase [49, 81, 82]. Taken together with the fact that Dex inhibited *Srd5a1* expression in GnRH neuronal cells, and 5α reductase affects GnRH synthesis, secretion, and pubertal timing [9], the glucocorticoid repression of *Srd5a1* that impacts reproductive function likely occurs in this part of the brain, although this gene is expressed other hypothalamic glial and neuronal cell types (data in [83]). Given that corticosterone is at a nadir already during late embryonic through perinatal development, *Srd5a1* levels in the POA are presumably high during this time, which is a period of exquisite POA sensitivity to gonadal steroids [84, 85] and when sexual dimorphism of the POA is established [85, 86]. Thus, even subtle changes in *Srd5a1* expression levels at this time might have important implications for early life development and later sexual maturation and reproductive function.

The effects of the glucocorticoid on *Srd5a1* transcription are likely mediated directly, given that GR is associated with the intronic enhancer in GnRH neuronal cells, though Dex treatment did not appear to increase GR binding at the enhancer, suggesting that the repression is mediated via GR interacting proteins and co-repressors. Although a consensus palindromic GR response element is not found in this genomic locus, a half site (GGGACA) reported previously to mediate monomeric GR repression of gene expression [87] is located at the region of enriched binding. GR actions are highly context-specific not only in terms of its DNA binding, but also in its mechanisms of activation, co-regulatory proteins, and the resulting outcomes [88, 89]. GR represses transcription of numerous genes, and at the *Crh* promoter, glucocorticoid-induced GR recruits DNMT3b to induce DNA methylation as well as recruitment of other proteins such as MeCP2 that are involved in the repression [81, 90]. However, DNA methylation at the intronic enhancer in the POA did not increase with (or before) the drop in *Srd5a1* levels, indicating that methylation at this site is not responsible for the drop in *Srd5a1* expression in the neonate.

In the ovary, the peripubertal stimulatory effect of E_2_ on *Srd5a1* appears to be dependent on low enhancer DNA methylation, which we found elevated following prepubertal immune challenge [9]. However, in the POA, the dominant regulation appears to be mediated by the hypothalamic-pituitary-adrenal axis and GR acting directly at the *Srd5a1* intronic enhancer. Although we did not detect reduced *Srd5a1* expression in the POA in the older mice, an age-related drop in 5α-reduced neurosteroids was reported recently [91] that might perhaps be due to the increase in glucocorticoid levels that occurs over the lifespan [92]. We have thus shown that this gene is regulated through cell-specific mechanisms, some of which help explain the long-term effects and particular sensitivity to stress experienced early in life [1, 9].

Guided by our earlier work, our study is limited in that it has focused on this one element of the early-life stress-response that is certainly complex and involves multiple cell types in both the POA and other tissues, encompassing 5α reductase-1-dependent and independent mechanisms. However, 5α reductase-1 is widely expressed, and its epigenetic regulation in other regions of the brain [93-96], as well as its role in catalyzing production of additional neurosteroids [11, 12, 14] have been reported, indicating multiple and diverse downstream effects on various endocrine systems. The epigenetic regulation of 5α reductase-1, together with recent indications of its role in pathologies such as polycystic ovarian syndrome and metabolic syndrome [14, 97], emphasize the need for further research to uncover more fully these and additional mechanisms underlying variation in reproductive phenotypes as influenced by early-life experiences, and also how such responses affect health across the life course.

## Data Availability

Some or all datasets generated during and/or analyzed during the current study are not publicly available but are available from the corresponding author on reasonable request.

## Abbreviations

ChiP: chromatin immunosuppression
dCas9: dead Cas9
Dex: dexamethasone
E_2_: estradiol
ERE: estrogen response element
FBS: fetal bovine serum
GR: glucocorticoid receptor
PND: postnatal day
POA: preoptic area
qPCR: quantitative polymerase chain reaction
TF: transcription factor
